# Birth preparedness and complications readiness among women in disadvantaged rural districts of Ghana

**DOI:** 10.1186/s12884-023-06041-2

**Published:** 2023-10-14

**Authors:** Abraham Rexford Oduro, Maria Anyorikeya, Patrick Ansah, Samuel Oladokun, Ernest Maya Tei, Randy Oduro-Ayeh, Paul Welaga, Seli Deh

**Affiliations:** 1 Research and Development Division, Ghana Health Service, Accra, Ghana; 2 Population and Health Research Centre, Dunkwa, Ghana; 3 Navrongo Health Research Centre, Navrongo, Ghana; 4 School of Public Health, University of Ghana, Legon, Accra, Ghana; 5 Krowor Municipal Health Directorate, Regional Health Directorate, Accra, Ghana

**Keywords:** Birth preparedness, Complication readiness, Rural districts, No hospitals, Ghana

## Abstract

**Introduction:**

Essentially all women and babies irrespective of their economic and social status should reach their full potential for health and well-being. The study assessed the readiness of mothers and their preparedness for birth across three disadvantaged rural districts in Ghana.

**Methods:**

A multi-centre quantitative survey from January to December 2018 using a multistage sampling approach was employed. Using a structured questionnaire data from mothers attending antenatal and postnatal clinics in three main ecological zones of Ghana were collected. Women who provided informed consent were consecutively recruited until the sample size was achieved. For categorical data, summary tables, proportions and percentage are presented. Multivariate logistic regression analysis determined the effect of selected characteristics on birth preparedness. Ethics approval was obtained from the Navrongo Health Research Centre.

**Results:**

A total of 1058 mothers were enrolled: 33.6%, 33.4% and 33.0% respectively from the Ada west, Upper Denkyira west and Builsa south districts. About 94% of the women had prior knowledge of birth preparedness. Approximately 22.6% (95%CI 20.1, 25. 2) of the mothers were assessed to have poor birth preparedness: 8.0% in Builsa south, 27.8% in Ada west and 31.7% in Upper Denkyira west. Prenatal and postnatal data showed no statistically significant difference in poor preparedness (21.9% vs 23.3%; *p*-value > 0.05). Maternal age, employment status, religious affiliation and parity were not associated with birth preparedness (*p*-value > 0.05). Area of study (*P* < 0.001), educational level (*P* < 0.016), marital status (*p* < 0.001) and antenatal contacts (< 0.001) were significantly associated with birth preparedness.

**Conclusions:**

As an important safe motherhood strategy woman should plan their pregnancy and birth well to reduce maternal and neonatal mortality. Policy initiatives should take into consideration area of residence, education, marital status and antenatal contacts of women.

## Introduction

Essentially all women and babies irrespective of their economic and social status should reach full potential for health and well-being. Although important progress has been made in the recent decades, maternal and neonatal mortality and morbidity remain unacceptably high especially in developing countries. Thus, ending preventable adverse maternal and neonatal outcomes remain at the top of the global health agenda [1–4]. Simply surviving pregnancy and childbirth is not enough marker of successful maternal health but critical efforts at promoting their well-being as a global health priority. Addressing inequalities in health interventions for mothers and babies is also fundamental to ensuring equity and high-quality care for them [1–4].

Safe motherhood initiatives have played key roles in birth plan strategies among expectant women and have helped to eliminate some of the delays associated with adverse outcomes [1–4]. There is however the need to redouble those strategies that improve birth preparedness and actions in case of emergency. Since it is impossible to predict which pregnancy will have obstetric emergency, it is important for women to be well prepared for the various scenarios that may arise. It is imperative for women to have contingency plans and resources in the event of an emergency [3–5].

In their birth planning, women are expected to organize the supplies and logistics they will need for delivery, decide on the location of the closet facility to give birth, the preferred birth attendant, identify a birth companion and funds for any expenses related to birth and in case of complications. They are also to make provision for transport, decide what they will do in an emergency, identify support to look after the home and other children while she is away. A good birth plan moderates any emergency that may occur during pregnancy and delivery and must be supported [6–10].

In Ghana, factors associated with the practice of birth preparedness and complication readiness have been documented but limited information exist in hard-to-reach communities where there are often no hospitals for emergency referrals [11–14]. This in addition to other factors contribute to making expectant mothers report late, unprepared for delivery and hence the associated poor outcomes. The contribution of this to the sustenance of the high maternal and neonatal mortality, and morbidity cannot be over emphasized.

Though maternal mortality ratio in Ghana have declined significantly in recent years. The pace of decline has slowed, and this might have led to its inability of Ghana to achieve the Millennium Development Goal target [15]. This is against the backdrop of the Ghana’s free maternal health policy that has been implemented since 2008 under the National Health Insurance Scheme [16]. The policy allows pregnant women to have free registration with the scheme to be entitled to free services throughout pregnancy, childbirth and postnatal [16]. The policy is one of the Ghana’s strategies for the achievement of the SDGs in reducing of maternal and child deaths and achievement of universal health coverage.

This study documented the practice of birth preparedness and complication readiness among pregnant women in three ecologically distinct settings in Ghana, where there are no district hospitals for immediate obstetric emergency referral care. The absence of a district hospitals often means that in Ghana women may have to travel for long distances before getting to the nearest district hospital and therefore if they have not prepared already, it will further worsen the delay in getting to the hospital. The study assessed the facteros that determine readiness of mothers and their preparedness for birth across three disadvantaged rural districts in Ghana.

## Methods

### Study area and setting

**Fig. 1:**
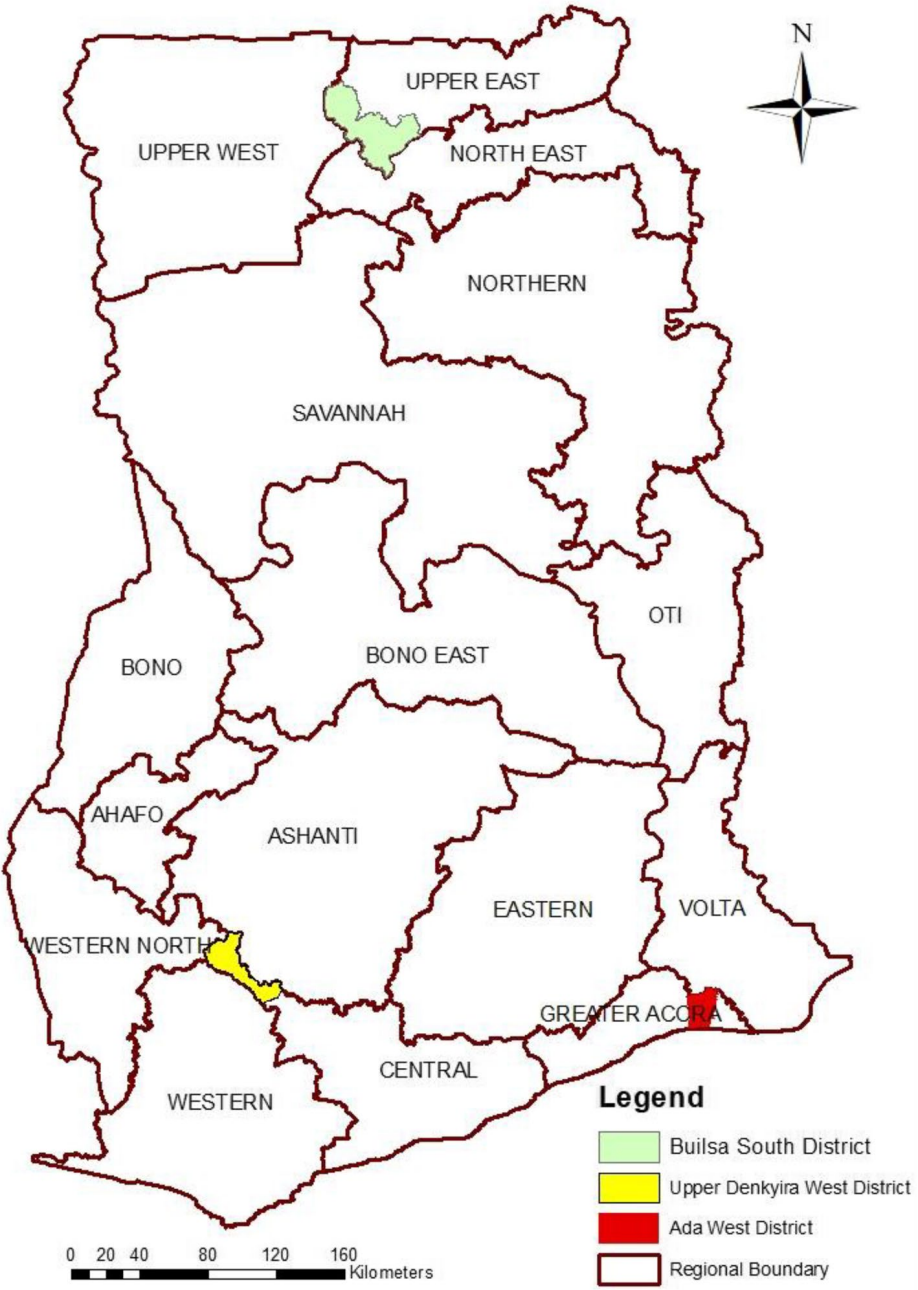
Map of Ghana showing the three administrative sites for the study

The study was coordinated in the Navrongo Health Research Centre [17] and data collected from three administrative districts in Ghana namely Ada West, Upper Denkyira West and Builsa South Districts (Fig. 1). These districts were chosen because they are all rural with no district hospitals for secondary level of care and their locations represent the three major ecological zones of coastal, forest and the savanna of the country (Fig. 1).

Among the three study areas upper Denkyira west is tha most economicall active lying in the middle zone of the country. It is a forest and cocoa growing area and within the gold mining area including the small scale and illegal ones. Builsa area is located in the northern part of Ghana where poverty is high, and vegetation is arid savanna and the Ada west though is in the south and closer to the national is economically less active.

The Ada West district lies between Latitudes 5°45’S and 6°00’N and Longitude 0°20’W and 0°35’E in the coastal savannah of Ghana with a land area of about 323.72 Square km.. The second district, Upper Denkyira west lies within latitudes 5º 30’ N and 6º 02’ N of the equator and longitudes 1º W and 2º W and has a total land area of 579.21 square kilometers within the semi-deciduous forest zone of Ghana (Fig. 1). The third district Builsa South lies between longitudes 1 0 05’ West and 10 35’ West and latitudes 100 20’ North and 100 50’ North of the equator in the guinea savannah parts of Ghana (Fig. 1). Physical access to health care services in the districts are limited by inadequacy of health facilities. Health care in the districts is delivered at two levels, the community and sub-district levels as none of the districts has a hospital. In addition to these facilities, mobile health teams visit several villages and communities on routine bases to offer health services in addition to private clinics [18].

### Study design

A cross-sectional survey with multistage sampling approach was conducted in the three study districts. A mixture of convenient and probability sampling approaches was used. The points of data collection included subdistrict antenatal and postnatal clinics.

The study duration was from January to October 2018.

Study population involved all pregnant women in their third trimester seeking antenatal care services and women who had delivered in the last six weeks in the three districts during the period of study. All those who met the selection criteria were recruited. Participants were enrolled consecutively until the sample size was achieved. Each person participated and provided information only once during the study period.

### Data collection

A structured questionnaire on birth preparedness was developed and used to obtain study information. The structured questionnaire was based on previous similar studies [9–14]. The questionnaire was prepared first in English and translated by fluent speakers into the local languages during interview. The questionnaire was pretested and piloted before deplored. After piloting, all suggested revisions were made before being administered in the actual study. The study variables involved were sociodemographic, reproductive history, obstetric factors, questions on birth preparedness and complication readiness. Data was collected through face-to-face interviews. Data collectors were recruited and paid to implement the study. Data collected were checked and reviewed on daily basis by supervisors before data entry.

Data collectors were university graduates from each district who spoke their local languages and were supervised by research supervisors who were senior health professionals in the respective study districts who also liaised with colleague staff in participating health facilities to facilitate and supervised data collection process. Supervisors and data collectors were all stationed in the study area throughout the data collection process. Research assistants who were first degree holders employed at the Navrongo Health Research Centre (NHRC) double checked completed forms and notified the project coordinator for completeness or otherwise.

**Fig. 2:**
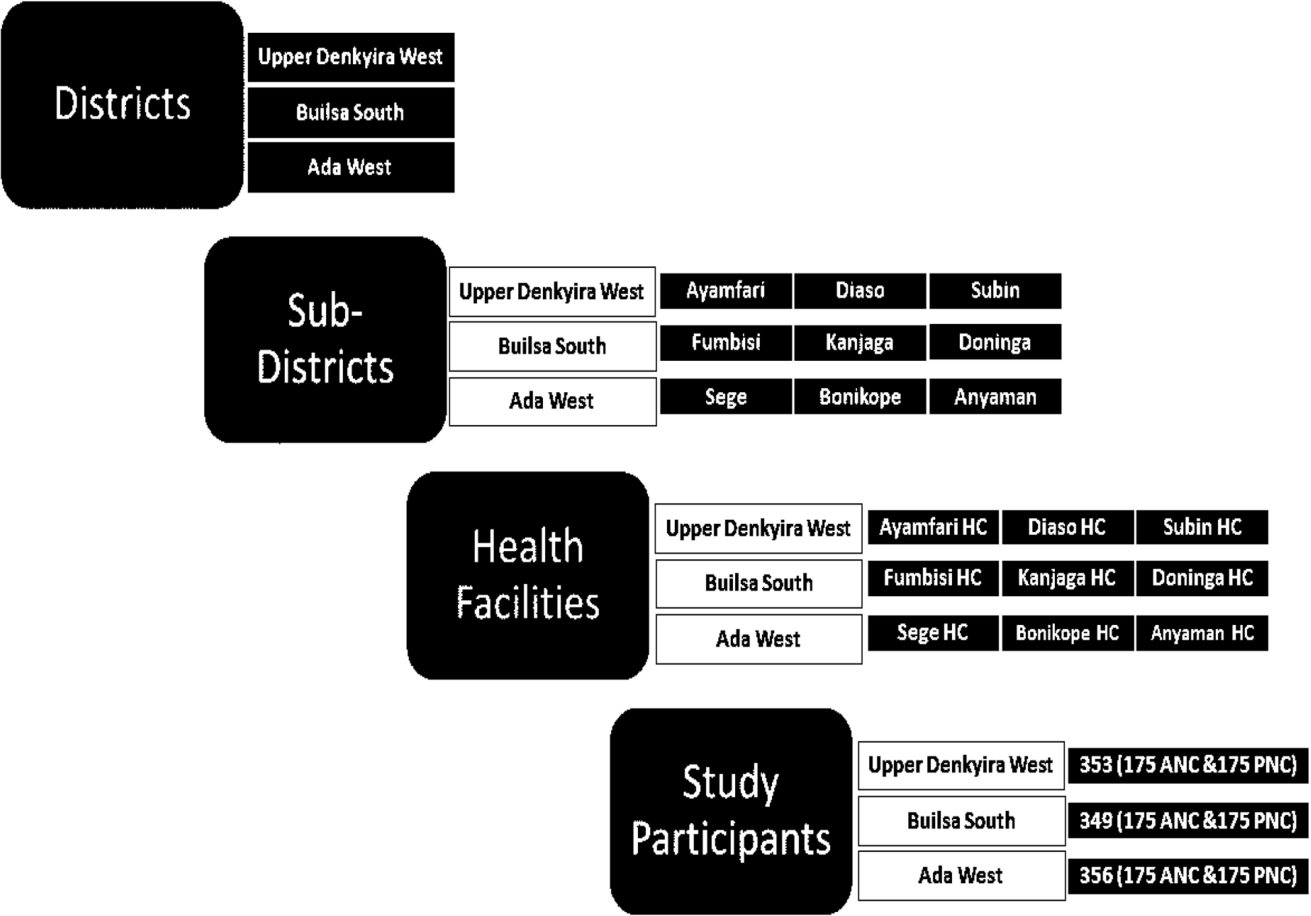
Sample frame showing each stage of the study sampling process

Sampling strategy and frame (Fig. 2). In each study district, three subdistricts were randomly selected and the largest antenatal clinic in each subdistrict chosen for the recruitment of participants. The total sample size for the district was shared among three subdistrict facilities by proportionate to size using the previous two months average client flow. For each subdistrict half of the participants were recruited from the mothers attending antenatal clinic and the other half from those attending postnatal clinic. Again, for each clinic, half of the women were enrolled from those having their first pregnancy and the half from those having their second or more pregnancy. For women who have just delivered in the last six weeks, half were women who have just delivered their first baby and the other half were women who have delivered their second or more baby. For cost and other reasons an estimated 1200 women for the study was determined and therefore 400 women per district. In each district, 200 pregnant women and 200 women who have delivered in the last six weeks were interviewed. Participants were consecutively recruited. Figure 2 shows the stages of the study sampling process.

### Sample size estimation

The sample size was calculated using a formula for estimation of single population proportion. An earlier study reported a level of birth preparedness to be 17%. A minimum sample size was obtained from each district using sample size calculation formula as shown below. Thus *n* = Z^2^ (p*q)/e^2^ *n* = 1.96^2^ (0.17*0.83)/0.05^2^ where: *n* = sample size, Z = percentile for 95% significance level for normal distribution (1.96) *P* = Prevalence of birth preparedness (17% = 0.17) *p* = 1-P e = margin of allowable error (0.05). With an alpha of 0.05 and a statistical power of 80%, 217 clients were targeted as minimum sample size per group. This sample size was adjusted to 250 to compensate for contingencies such as non-response, recording errors and to allow for generalization of results.

### Quality assurance

Training was given to the supervisors, research assistants and data collectors at the districts on how to administer the questionnaire and translate technical terms into local language for the understanding of participants. Data collectors were taught on how to ensure that the dignity and rights of participants were respected and on how to seek consent from all participants before questionnaires were administered. They ensured completeness and consistency of information during data collection. The questionnaire was pretested in one clinic and the study piloted before the main activities were made to commence. This ensured that all misunderstandings, ambiguities and inconsistencies were corrected. The research supervisor checked filled questionnaires from each data collector and meetings held during the data collection process for improvement.

### Data management and analysis

The collected data was reviewed, coded and double entered. Data obtained was initially entered into Epi-data, verified and cleaned. This was then exported into STATA for analysis. All data where necessary were coded using numeric values. For continuous variables, summary tables of means, standard deviations and ranges are presented. For categorical data, summary tables, proportions and percentage are presented. Bar chart was also used to depict the data. Bivariate analyses using Chi square test and logistic regression were undertaken to determine association of effect of selected characteristics on birth preparedness. Those found significant (*p*-value ≤ 0.05) were entered in the multivariable logistic regression analysis. The results are presented in frequency tables, odds ratio and 95% confidence interval. *P*-value < 0.05) denote statistical significance.

## Study results

### Background characteristics

A total of 1058 mothers consented and enrolled into the study. Approximately 33.6% were from the Ada west district, 33.4% from the Upper Denkyira west district and 33.0% from the Builsa south district of Ghana. About 50.6% of the mothers were recruited in the antenatal clinics and 49.4% in the postnatal clinics. The average maternal age was 26.7 (Range; 14–49) years. The average age in years for Ada west was 26.9 (95% CI 26,4, 27.2), Upper Denkyira west was 26.9 (95% CI 26.6, 27.1) and Builsa south was 26.6 (95% CI 26.2, 29.9). The average age was 27.2 (95%CI 26.7, 27.5) and 26.4 (95%CI 26.0, 26.7) years for the antennal and postnatal mothers respectively. In all 10.8% of the mothers were under 20 years, 64.6% between 20 to 30 years and 24.6 above thirty years. The proportion of mothers under 20 years was lowest in Upper Denkyira west 2.9% (95%CI 1.3, 5.1) compared to 13.3% (95%CI 9.9,17.2) in Ada west and 16.4% (95% CI 12.7, 20.8) in Builsa south (Table 1). Some background characteristics showed some differences among the three study sites.

**Table 1:** Socio-demographic characteristics of the study participants by district

Characteristics	Categories	Study District, n (%)			
		**Ada West**	**Denkyira West**	**Builsa South**	**Total**
**Maternal Status**	Prenatal	185 (52.0)	176 (49.9)	174 (49.9)	535 (50.6)
	Postnatal	171 (48.0)	177 (50.1)	175 (50.1)	523 (49.4)
**Maternal Age**	< 20	47 (13.3)	10 (2.9)	57 (16.5)	114 (10.9)
	20–30	215 (60.7)	266 (76.0)	197 (56.9)	678 (64.6)
	> 30	92 (26.0)	74 (21.1)	92 (26.6)	258 (24.6)
**Education Level**	None	78 (22.0)	71 (20.2)	106 (30.7)	255(24.3)
	Primary	223 (62.8)	229 (65.3)	201 (58.3)	653 (62.1)
	Secondary	54 (15.2)	51 (14.5)	38 (11.0)	143 (13.6)
**Marital status**	Single	93 (26.2)	35 (9.9)	6 (1.7)	134 (12.7)
	Married	248 (69.9)	230 (65.4)	34 0 (97.7)	818 (77.5)
	Others	14 (3.9)	87 (24.7)	2 (0.6)	103 (9.8)
**Employment status**	None	100 (28.3)	52 (14.8)	173 (49.6)	325 (30.8)
	Employed	39 (11.0)	21 (6.0)	16 (4.6)	76 (7.2)
	Self employed	215 (60.7)	278 (79.2)	160 (45.8)	653 (62.0)
**Religious affiliations**	Christianity	328(94.0)	298(85.9)	299(86.4)	925 (88.8)
	Islam	15(4.3)	40(11.5)	29(8.4)	84 (8.1)
	Others	6(1.7)	9(2.6)	18(5.2)	33 (3.2)
**Number of previous Pregnancies**	One	97(27.5)	14(3.9)	106(30.4)	217 (20.6)
	Two	89(25.2)	37(10.5)	77(22.0)	203 (19.2)
	> two	167(47.3)	302(85.6)	166(47.6)	635 (60.2)
**ANC attendance**	Once	33(9.3)	3(0.9)	2(0.7)	38 (3.6)
	Twice	45(12.6)	13(3.7)	18(5.2)	76 (7.2)
	Three times	63(17.7)	22(6.2)	34(9.7)	119 (11.2)
	Four times	77(21.6)	72(20.4)	55(15.7)	204 (19.3)
	Four +	138(38.8)	243(68.8)	240(68.7)	621 (58.7)

About 24.3% of the participants had no formal education, 62.1% had basic education and 13.6% had secondary education and above. Approximately 77.5% of the participants were married, 12.7% were single and 9.8% either divorced, separated or widowed. Single motherhood was least among the Builsa south participants 1.7% (95% CI 0.6, 3.7) compared to 9.9% (95%CI 7.0, 13.5) in Upper Denkyira west and 26.2% (95%CI (21.6, 31.0) in Ada west districts. On employment status, about 30.8% of the women indicated they were unemployed, about 62% reported self-employment and only 7.2% had formal employment. Participants from the Builsa district had the highest unemployment rate 49.6% (95%CI 44.2, 54.9) compared to those from Upper Denkyira west 14.8% (95% CI 11.2,18.9) and Ada west 28.3% (95%CI 23.6, 33.2). Majority of the participants were Christians 88.8% and Moslems were 8.1% and other faiths accounted for 3.1%.

In terms of pregnancy, 20.6% of the women were gravida one, 19.2% were gravida two and 60.2% reported had gravida three or more. For women who had more than two pregnancies, those from Upper Denkyira west district had the highest 85.6% (95%CI 81.4, 89.0) compared to those in Ada west 47.3% (95%CI 42.0, 52.9) and Builsa south 47.6% (95%CI 42.2, 52,6). In all about 41.3% had four or less contacts and 58.7% had more than four antenatal contacts (Table 1).

### Knowledge of birth preparedness

Table 2 presents participants responses in relation to their birth preparedness by district. Overall, 94% indicated having heard about birth preparedness before; Builsa south district had 99.4%; Ada west 89.9% and Upper Denkyira west 92.6%. In all only about 39% of respondents were aware of their expected date of delivery. This varied from Upper Denkyira west (56%) and Ada west (52.3%) compared to the 9% (95%CI 6.3,12.8) in Builsa south.

**Table 2:** Participants responses to their birth preparedness across the three districts

**Questions to participants on birth preparedness**	**% (95% CI)**			
	**Ada West**	**Denkyira West**	**Builsa South**	**Total**
Heard about birth preparedness before	89.9 (86.3, 92.8)	92.6 (89.4, 95.1)	99.4 (97.9, 99.9)	94.0 (92.3,95.3)
Know the expected date of delivery	52.3(46.9, 57.5)	56.0 (50.6, 61.2)	9.2(6.3,12.8)	39.3(36.3,42.4)
Aware labor may start before the due date	71.6(66.6,76.2)	92.9(89.7, 95.4)	40.5(35.3, 45.9)	68.5(65.5,71.3)
Made provision during day of labor for transport	68.6(63.5, 73.4)	30.9(26.0, 36.0)	83.6(79.0, 87.1)	61.0(57.9,63.9)
Made provision for transport for night labor	66.0(60.7, 71.0)	27.9(23.3,32.9)	83.4(79.0, 87.1)	59.0(55.9, 62.0)
Had saved money for hospital expenses	73.3(68.3, 77.8)	70.8(65.6,75.5)	80.8(76.2, 84.8)	75.0(72.2, 77.5)
Made provision for referral to another hospital	21.9(17.6, 26.6)	12.5(9.2,16.4)	4.0(2.2, 6.6)	12.8(10.8, 15.0)
Indicated she lacked financial support	20.3(16.2,24.9)	67.1(62.0,72.0)	95.4(92.7, 97.4)	60.8 (57.7, 63.7)
Have national health insurance	98.0(93.4, 97.8)	100.0 (0, 0)	100.0 (0, 0)	98.7(97.7,99.3)
Health insurance renewed for the year	87.8(83.9, 91.0)	97.5(95.2, 98.8)	69.9(64.8, 74.7)	85.1(82.8, 87.2)
Identified a place of delivery	90.1(86.4, 93.0)	89.0(86.1, 93.0)	99.7(98.4, 100)	93.2(91.4,95.6)
Identified birth companion during delivery	94.4(91.4, 96.5)	94.3(91.3, 96.5)	98.9(97.0, 99.7)	95.8(94.4,97.0)
Have identified a possible blood donor	35.1(30.1, 40.4)	10.7(7.6,14.4)	91.1(87.6,93.9)	45.6(42.5,48.7)

A total of 68.5% (721) of respondent were aware that labor may start before due date. Only 40.5% (95%CI 35.3, 45.9) respondent in the Builsa area knew their labour may start before the due date compared to 93% (95%CI 89.7,95.4) in the Denkyira and 72% (66.6, 76.2) in Ada districts.

On the question of making provision for transport during the day of labour, 61.0% answered in affirmation; a relatively higher percentage in the Builsa 83.6% (95%CI 79.0,87.1) and in Ada 68.6% (95%CI 63.5,73.4) compared to Denkyira 30.9% (95%CI 26.0, 35.0). Similar estimate (59.0%) was found for night transportation (Table 2). About 75% of the women reported saving money for hospital expenses during their pregnancy. More in women in the Builsa area (80.8%) compared to those in the Upper Denkyira west (70.7%) and Ada west (73.3%).

Overall fewer women (12.8%) made provision for referral to another hospital should it arises. This ranged from 21.9% (95%CI 17.6,26.6) in Ada west to 4.0% (95%CI 2.2, 6.6) in the Builsa south district and 12.5% (95% CI 9.2,16.4 in the Upper Denkyira west. Overall, 60.8% of the women reported that they lacked financial support during their pregnancy. This ranged from Builsa south district (95%) and Upper Denkyira west (67%) compared to 20% in the Ada west (Table 2). Approximately 98.7% of the women indicated they have registered with the national health insurance scheme and 85.1% had renewed their insurance at the time of the study with those Builsa south (70%) less likely to renew their insurance compared to those from Ada west (88%) and Upper Denkyira west (97%).

From the results 93.2% of the women had identified a place of delivery while 95.8% had identified a companion to the hospital during labour. On whether they had identified blood donor in the event blood is needed a total of 45.6% indicated in affirmation and this varied widely from 91.1% from the Builsa south district to 35.1% in the Ada west district and the Upper Denkyira west district recording a very low (10.7%) Table 2.

### Determinants of birth preparedness

**Fig. 3:**
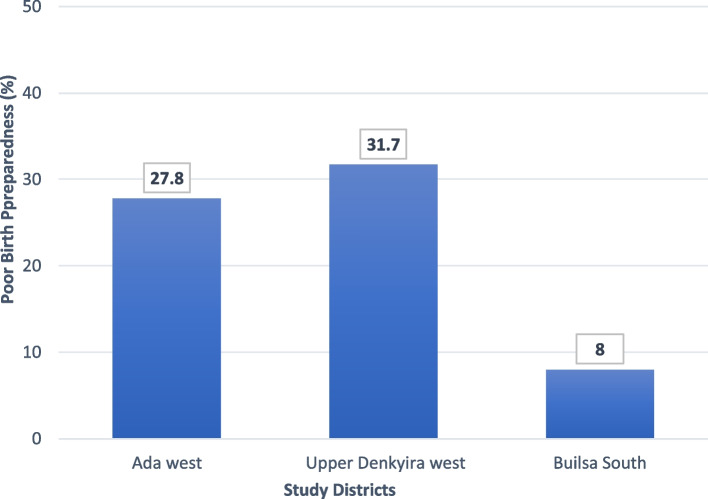
Proportion of women with poor birth preparedness in the three study districts

Using the thirteen questions for the study questionnaire (Table 2), we estimated the proportion of the participants who had less than average (poor) birth preparedness. This applied to all the participants who had less than average score among the study participants. In all 22.6% (239/1058) of the women deemed to have poor birth preparedness compared with 77.4% (819/1058) deemed to have good preparedness to. Among those with poor preparedness, the Builsa south district had the lowest value 8.0% (95%CI 4.8, 12.1) and this was significantly lower than Ada west 27.8% and Upper Denkyira west 31.7% Fig. 3.

There was no statistical significance (*p*-value > 0.05) between the poor birth preparedness of mothers attending prenatal clinics (21.9%) and mothers attending postnatal clinics (23.3%).

Maternal age was not associated with poor preparedness (*p*-value = 0.3). However, mothers below twenty years had the highest poor preparedness (27.2%), this was not statistically different from those between 20 to 30 years (21.2%) and those above 30 years of age (23.6%), Table 3. From the results of the study, the higher the maternal level of education the lower the proportion of poor preparedness. Mothers who had not been to school had 29.4% poor preparedness compared to those with basic education (21.6%) and secondary or more education (16.1%) *p*-value < 0.005). Further, single mothers (37.3%) were less prepared for birth compared to married mothers (17.7%) and this was very statistically significant (*p* < 0.001). The employment status of the women interviewed influenced their level of poor preparedness. Whereas 10.5% of the mothers who had formal employment had poor birth preparedness, those with no employment and self-employment had 21.5% and 24.5% poor preparedness respectively Table 3.

**Table 3:** Level of birth preparedness and complication readiness by baseline characteristics

Characteristics	Categories	Frequency	Poor Preparedness	Statistics
		n (N)	% (95CI)	*p*-value
**Districts**	Ada west	99 (356)	27.8 (22.0, 33.8)	< 0.001*
	Denkyira west	112 (353)	31.7 (25.9, 38.1)	
	Builsa South	28 (349)	8.0 (4.8, 12.1)	
**Maternal Status**	Prenatal	117 (535)	21.9 (16.7, 27.5)	0.571
	Postnatal	122 (523)	23.3 (18.2, 29.3)	
**Maternal Age**	< 20	31 (114)	27.2 (21.5, 33.3)	0.324
	20–30	144 (678)	21.2 (16.1, 27.0)	
	> 30	61 (258)	23.6 (18.4, 29.7)	
**Education Level**	None	75 (255)	29.4 (23.5. 35.5)	0.005*
	Primary	141 (653)	21.6 (16.7, 27.5)	
	Secondary +	23(143)	16.1 (11.5, 21.2)	
**Marital status**	Single	50 (134)	37.3 (31.2, 43.9)	< 0.001*
	Married	146 (818)	17.9 (13.0, 23.1)	
	Others	42 (103)	40.8 (34.4, 47.3)	
**Employment status**	None	70 (325)	21.5 (16.3, 27.2)	0.019*
	Employed	8 (76)	10.5 (6.9, 15.1)	
	Self employed	160 (653)	24.5 (19.0, 30.3)	
**Religious affiliations**	Christianity	205 (925)	22.2 (16.9, 28.0)	0.680
	Islam	22 (84)	26.2 (20.8, 32.5)	
	Others	8 (33)	24.2 (18.9, 30.3)	
**Pregnancies**	One	42 (217)	19.4 (14.4, 24.8)	0.233
	Two	42 (203)	20.7 (15.5, 26.2)	
	> two	155 (635)	24.4 (18.9, 30.2)	
**ANC attendance**	Once	25 (38)	65.8 (59.3, 71.7)	< 0.001*
	Twice	31 (76)	40.8 (34.3, 47.1)	
	Thrice	32 (119)	26.9 (21.2, 32.9)	
	Four times	56 (204)	27.5 (22.0 33.8)	
	Four +	95 (621)	15.3 (11.1 20.7)	

Overall, the results indicate that the number pregnancies a woman has had and her religious affiliations did not determine her level of poor preparedness Table 3. Though Christian mothers (22.2%) had the lowest poor preparedness compared to Moslem mothers (26.2%) and other Faiths (24.2%), this did not reach statistical significance. Surprisingly mothers who reported more than two previous pregnancies (24.4%) were the highest unprepared compared to those with two (20.7%) and one (19.4%) pregnancy respectively, Table 3.

**Fig. 4:**
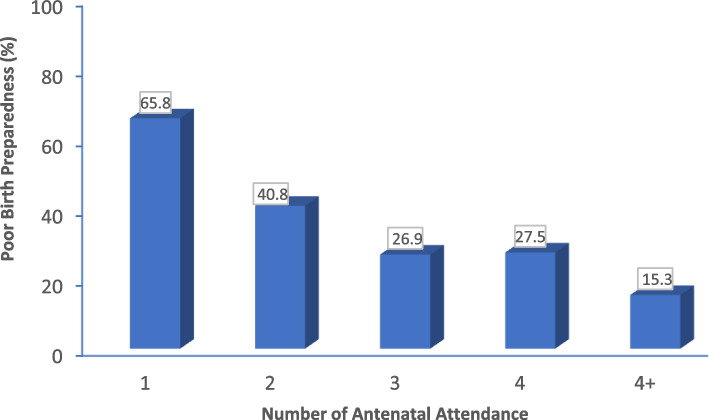
Relationship between antenatal clinic contacts and poor birth preparedness

The results showed a negative correlation between the number of antennal contacts and the level of birth unpreparedness of the women Fig. 4.

Using logistic regression and adjusting for the background characteristics, the area of study (*P* < 0.001), educational level (*P* < 0.016), marital status (*p* < 0.00) and antenatal attendance (< 0.001) were the factors associated with birth preparedness (Table 4).

**Table 4:** Determinants of birth preparedness in three rural districts of Ghana

Factors	Unadjusted		Adjusted	
	**OR (95% CI)**	***p*-value**	**OR (95% CI)**	***p*-value**
**Districts**				
Ada west	1	< 0.001	1	< 0.001
Denkyira west	1.20(0.87 1.65)		1.80(1.15 2.82)	
Builsa south	0.23(0.14 0.36)		0.33(0.20 0.57)	
**Maternal Age**				
< 20	1		1	
20–30	0.72(0.46 1.13)	0.326	0.55(0.28 1.07)	0.080
> 30	0.83(0.50 1.37)		0.74(0.34 1.63)	
**Education Level**				
None	1		1	
Primary	0.66(0.47 0.92)	0.005	0.57(0.38 0.84)	0.016
Secondary	0.46(0.27 0.77)		0.54(0.29 1.02)	
**Marital status**				
Single	1		1	
Married	0.37(0.25 0.54)	< 0.001	0.63(0.38 1.05)	0.001
Others	1.16(0.68 1.96)		1.56(0.83 2.95)	
**Employment status**				
None	1		1	
Employed	0.43(0.20 0.93)	0.024	0.41(0.16 1.04)	0.162
Self employed	1.18(0.86 1.63)		0.92(0.59 1.43)	
**Religious affiliations**				
Christianity	1		1	
Islam	1.25(0.75 2.07)	0.6808	1.19(0.67 2.11)	0.836
Others	1.12(0.78 0.50)		1.09(0.41 2.92)	
**Pregnancies**				
One	1		1	
Two	1.08(0.67 1.75)	0.234	1.17(0.62 2.21)	0.8393
> two	1.35(0.92 1.97)		1.20(0.64 2.27)	
**ANC attendance**				
Once	1		1	
Twice	0.36(0.16 0.81)	< 0.001	0.33(0.13 0.82)	< 0.001
Three times	0.19(0.09 0.42)		0.18(0.08 0.44)	
Four times	0.20(0.09 0.41)		0.17(0.07 0.39)	
Five +	0.09(0.05 0.19)		0.09(0.04 0.20)	

## Discussion

As a component of focused antenatal care, birth preparedness is an important safe motherhood strategy intended to help women and families plan for safe pregnancy and childbirth to help reduce maternal and neonatal mortality. This study determined the level of birth preparedness in three disadvantaged rural districts of Ghana and examined the associated factors. The results showed that fewer women were unprepared for delivery. Only one out of every four mothers had less than average preparation, of which four factors; area of study, educational level, marital status and the frequency of antenatal contacts were found to be significantly associated with them.

### Educational attainment

The results show that level of maternal educational attainment was significantly associated with birth preparedness. Women with primary or secondary education were twice likely to be prepared for their delivery compared to those without education. However, there was no significant difference between those with primary and secondary education. The positive relationship between maternal education and birth preparedness in this study is consistent with others documented in Ghana and in other sub-Saharan countries [19–22]. This positive effect may be explained by the fact that women who have higher education are more knowledgeable about obstetric danger signs and adverse birth outcomes. Such educated women are more inclined to utilize essential health services, access skilled delivery attendance and prepare well to avert delays during labour [19–22]. In a study among expectant mothers in northern Ghana, it was observed that as the educational level of these mothers increased, there was a corresponding increase in the level of birth preparedness, with women having at least primary education twice more likely to deliver at facility compared to their counterparts with no formal education [12]. Furthermore, as women climbs up the educational ladder, they are likely to become financially more empowered with much disposable income that enables them to either own their personal means of transport or make arrangement in order to access skill delivery at farther distances from their place of residence [19–22] [24]. Other studies have also found the lack of association of maternal level of education as these women may have ignored their own knowledge of reproductive health and relied mainly on the advice of their partners in decision making, a practice very common in patriarchal African society, [13, 19–22].

### Antenatal contacts

The study showed a significant positive correlation between the number of antennal contacts a woman has during her pregnancy and the level of birth preparedness. Antenatal contact is a preventive health care that provides women the opportunity for early facility-based assessment. Its importance is dependent on the adequacy of the content, timing, frequency and quality of health education during ANC sessions. Our result is consistent with an earlier finding in Ghana and thus affirms the importance of ANC as a predictor of birth preparedness, in Ghana [14]. This thus underscores the need for continuous adherence to antenatal care protocols in Ghana and elsewhere to ensure that women are adequately educated for a positive pregnancy experience. There is variability of evidence on the effectiveness of timing of ANC visits on birth preparedness [2, 12, 19–22]. Some studies suggest increased likelihood of birth preparedness among women who attend ANC early while other evidence suggest women who initiate ANC at late gestational age are more likely to be birthprepared. On the frequency of ANC visits, four or more visits have reportedly been linked with increased awareness of danger signs. Recent evidence from WHO recommend frequent antenatal contacts of eight or more is associated with a reduced likelihood of adverse birth outcomes due to increased opportunities to detect and manage potential complications [2, 12, 19–22].

### Marital status

Married women and those with other status were found to be significantly associated birth preparedness. This significant determinant of birth preparedness in this study is similar to findings from studies elsewhere [19–22]. This finding has been explained to be attributable to the social and economic support that married women enjoyed. Sometimes pregnancies outside of a union of marriage is culturally frown upon in our setting and as such unmarried women tend to shy away from public view and less amenable to discuss issues of maternal health [19–22]. Moreover, married women are likely to be better placed financially to pay for the cost of facility delivery even at distant places and therefore likely to seek skill delivery and also likely to enjoy spousal and related support [19–22].

### Study area

On the association of area of study to birth preparedness among women, of the three areas of the study, participating women from the Builsa south district were the most prepared for birth. Several findings from the study might have accounted for this outcome. The proportion of mothers under 20 years of age was highest in Builsa South district compared to the other two districts whilst single motherhood was least among the Builsa south participants even though about half of the participants from the Builsa district were unemployed compared to the other participants. This could have contributed for this finding. Our findings already showed that mothers with fewer pregnancy who are likely to be young have higher preparedness. And also, being likely to be married means that they also enjoy spousal support. While this finding was to do with study area other studies have however found important association between birth preparedness and area of residence. Women who reside in urban areas have been documented to be more prepared for birth than those in rural areas. The availability and proximity to health facility affect utilization of health care including maternal health services. It is expected that pregnant women are more likely to access antenatal services and improve on their knowledge of birth preparedness when they are at close proximity to a health facility and staff [19–22].

Limitations of the study; this study is limited to only three rural districts in Ghana and looks only at areas without district hospital which may limit its representativeness. Again, without a qualitive component the study does not provide insights that are context and setting specific to help explore the reasons why we have these findings.

## Conclusions of the study

The study concluded that area of study, educational level, marital status and antenatal contacts were significantly associated with birth preparedness in our setting. Thus, as an important safe motherhood strategy woman should plan their pregnancy and birth to reduce mortality. Policy initiatives for improved maternal health should also take into consideration area of residence, maternal educational, marital status and antenatal contacts as critical factors for improved health.

## Data Availability

The datasets used and/or analysed during the current study are available from the corresponding author on reasonable request.

## Abbreviations

ANC: Antenatal Care
CI: Confidence Interval
HC: Health Centre
MGD: Millennium Development Goals
NHRC: Navrongo Health Research Centre
OR: Odds Ratio
PNC: Postnatal Care
SDGs: Sustainable Development Goals
WHO: World Health Organization
